# Correcting the “light-diet default”: nutrient density gaps in hospital-based postpartum nutrition services in China and system-level responses

**DOI:** 10.3389/fpubh.2026.1769297

**Published:** 2026-04-10

**Authors:** Qiaolin Liu, Jiali Liu

**Affiliations:** 1 Department of Adult Critical Care Nursing, West China Second University Hospital, Sichuan University, Chengdu, SichuanChina; 2 Key Laboratory of Birth Defects and Related Diseases of Women and Children (Sichuan University), Ministry of Education, Chengdu, China

**Keywords:** China, health system design, hospital food services, light diet default, maternal recovery, nutrient density, postpartum nutrition, quality indicators

## Abstract

Postpartum hospital stays in China are typically brief and highly standardized, yet they coincide with active tissue repair and, for many mothers, lactation initiation—both of which increase requirements for energy, protein, and micronutrients. In many hospitals, a culturally familiar “light diet” is adopted as the default because it is perceived as safe, well tolerated, and operationally efficient; however, when explicit nutrient specifications are absent, “light” can function as a non-clinical label that permits low nutrient density and underprovision of protein, energy, and foods that deliver key micronutrients. This Perspective examines how the “light-diet default” is produced across the care pathway—from ambiguous labeling and procurement-driven simplification to shortened menu cycles, default order sets, and monitoring practices that prioritize delivery consistency over nutritional adequacy—and argues that low nutrient density should be treated as a modifiable exposure rather than a neutral preference. Drawing on recovery-oriented postoperative nutrition principles and healthcare food-governance approaches, we propose a system-level correction that resets the default to “nutritionally adequate light.” The proposed approach defines a minimum standard for postpartum meal trays, integrates dietitian input into menu specifications and exception pathways, and embeds a concise, auditable indicator set (default integrity, tray compliance, exception rate with reasons, intake proxy, and patient experience) into routine governance and contracting. Correcting defaults—rather than rejecting “light” eating—can preserve cultural acceptability while making adequacy measurable, accountable, and continuously improvable in routine postpartum care.

## 1 Introduction

### 1.1 Postpartum hospitalization as a critical nutrition window

Postpartum recovery coincides with active tissue repair and, for many mothers, lactation initiation—both of which increase nutritional requirements relative to the non-pregnant state (1). Without reproducing full modeling assumptions, it is sufficient here to note that the Nordic Nutrition Recommendations 2023 treat lactation as a substantive increment in maternal needs—on the order of approximately 2 megajoules per day of additional energy (with some contribution from physiologic mobilization of maternal stores) and ~10–20 g/day of additional protein, depending on postpartum stage (1). In China, postpartum length of stay is typically short and ward meals are highly standardized; consequently, even modest shortfalls in protein–energy delivery (and in foods that carry key micronutrients) can recur across multiple trays within a narrow recovery window (2, 3). Postpartum hospitalization, however, is not solely a biomedical recovery episode; it is also a brief period of rest, bonding, feeding establishment, and transition to newborn care. The argument here is therefore not that every individual tray is clinically decisive, but that, during a short and highly standardized stay, mothers should not need to negotiate for nutritionally adequate options while managing the competing demands of early recovery. This framing positions ward menus as a modifiable care environment: postpartum nutrition can be engineered as part of clinical delivery through menu standards, default order sets, escalation rules, and monitoring, rather than treated as a catering add-on (4). Enhanced recovery recommendations after cesarean delivery further support early oral intake and timely diet advancement when clinically appropriate, reinforcing the feasibility of standardized postpartum nutrition workflows in routine practice (5, 6).

### 1.2 The emergence of a “light-diet default” in hospital practice

In many Chinese postpartum inpatient settings, a “light diet” functions as a culturally familiar, low-dispute default rooted in recovery-oriented eating scripts rather than in a formal nutritional standard. In this context, “culturally familiar” refers specifically to Chinese postpartum care traditions that often favor foods perceived as warm, gentle, and easy to digest, such as soups, porridges, soft staples, low-fat preparations, and minimally seasoned dishes. This does not imply uniform practices across all regions, hospitals, or mothers; rather, it identifies a recurrent recovery-oriented pattern that is often simplified into a generic “light” category within standardized inpatient food service. When explicit nutrient targets are absent (e.g., minimum daily protein, energy adequacy, and micronutrient density), however, “light” becomes a non-specific label that permits interpretation drift across procurement constraints, kitchen practices, and clinical nutrition delivery (4). A food-as-medicine framing therefore argues that integrating nutrition into healthcare requires measurable standards and workflow-linked actions across both clinical and food-service systems, rather than reliance on generic diet labels alone (4).

In practice, hospital food-service models may vary, including differences in menu ordering, kitchen arrangements, and the degree of bedside choice. The recurring issue addressed here, however, is not a single national service model but the operational conversion of an ambiguous “light” label into a standardized inpatient category. Once embedded in preset menu cycles, order sets, and routine tray delivery, “light” can become a low-exception, easily deliverable default unless nutrient specifications, dietitian-defined criteria, and explicit exception pathways are built into the workflow. The concern is therefore less where food is prepared than how menu logic, ordering structure, and service governance determine what is routinely delivered to postpartum mothers.

### 1.3 Author position and purpose of this article

This article is a systems-oriented Perspective written from the standpoint of authors working as obstetric clinical nurses within a Chinese maternal-care context. It is intended to identify a service-design problem and propose an audit-ready framework for improvement, rather than to provide a national survey of hospital menus or a formal evidence review.

## 2 Evidence base informing this perspective

This Perspective is informed by published literature and publicly available guidance relevant to postpartum inpatient nutrition, post-cesarean recovery, hospital food-service operations, menu governance, and patient meal experience. We consulted major health and nursing databases and also reviewed reference lists and practice-facing guidance documents to identify sources that could contextualize the argument. These materials were used to support a conceptual, practice-oriented synthesis rather than to conduct a formal systematic or scoping review. For clarity, the discussion is organized around four practical dimensions relevant to the service pathway: cultural scripts, operational constraints, adequacy targets, and governance levers. These dimensions were used as a pragmatic scaffold for synthesis, aligned with the article’s service-pathway logic, rather than as outputs of a formal coding process.

## 3 How the “light-diet default” is produced

Postpartum food expectations in China are shaped by recovery scripts that privilege foods framed as warm, gentle, and easy to digest, alongside avoidance logics and lactation-related beliefs (7). Urban-focused accounts of postpartum customs suggest these norms remain influential, rendering “light” a culturally legible marker of appropriate care (7). Comparative evidence on postpartum dietary patterns among Chinese women likewise notes frequent reliance on soups and soft staples, which translate readily into institutional menu categories and ordering labels (8). When “light” functions as a prudence cue without explicit nutrient targets, it operates less as a clinical prescription than as a permissive category that can accommodate wide variation in actual nutrient delivery.

Hospitals then translate cultural prudence into operational prudence. “Safety” is often implemented as minimizing gastrointestinal intolerance, perceived complications, and complaints during brief postpartum stays (5). Conservative menus reduce tolerance variability and simplify bedside counseling in high-throughput wards (5). Yet enhanced recovery recommendations after cesarean delivery support early oral intake and timely diet advancement when clinically appropriate (5). This creates a practical tension: day-to-day risk minimization can dominate menu design even when patients are clinically stable and recovery pathways support progression.

Food-service governance can further amplify conservatism through standardization and contract logic. Healthcare food standards emphasize procurement specifications and auditable compliance, which can favor predictable, low-variance items that are easy to produce and verify across shifts (9, 10). When combined with centralized menu cycles and staffing constraints, this approach can narrow offerings to those that best satisfy deliverables with minimal exceptions (9). Mixed methods research similarly indicates that hospital and long-term care food environments are shaped by organizational policy and commercial arrangements, not solely by individual preference (11).

A final mechanism is the conversion of an ambiguous label into an “orderable default.” When ordering interfaces define “light” without nutrient targets (e.g., minimum protein per tray or fortified options), the system cannot distinguish a gentle diet from an underpowered one (9). If order sets and menu cycles repeatedly route patients into the same category, the default becomes self-reinforcing. If monitoring prioritizes delivery key performance indicators and satisfaction over nutrient adequacy, improvement efforts predictably optimize consistency rather than composition.

**Figure 1:**
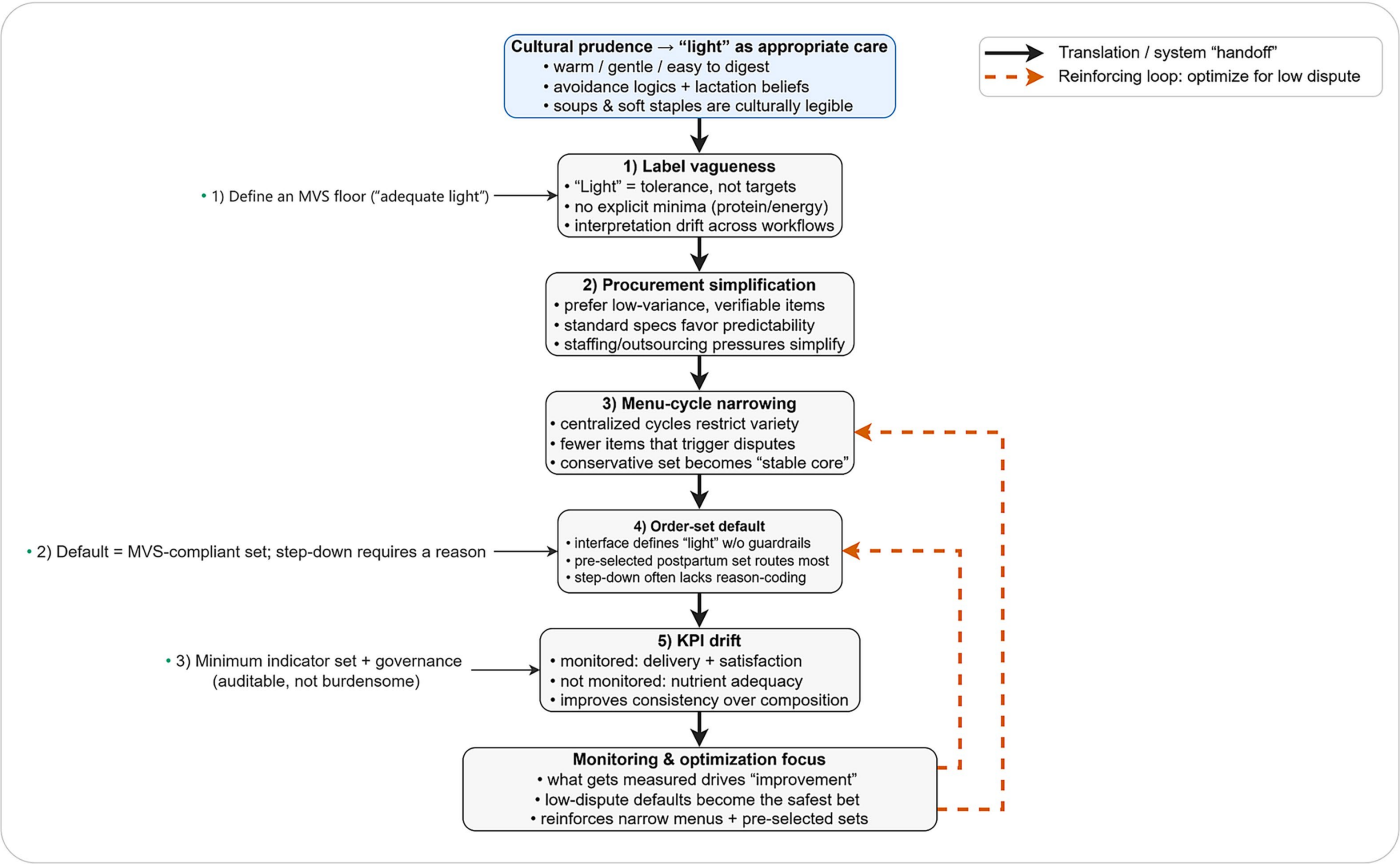
How an ambiguous “light-diet default” is produced in postpartum hospital food services, and where it can be corrected. This conceptual diagram summarizes how cultural prudence makes “light” a low-dispute marker of appropriate care, but label vagueness allows interpretation drift when nutrient targets are not explicit. The label is then operationalized through procurement simplification, menu-cycle narrowing, and an order-set default, shaping what is routinely delivered. When monitoring emphasizes delivery and patient satisfaction over nutrient adequacy, KPI drift shifts “improvement” toward consistency rather than composition and can reinforce the default (dashed arrows). Numbered callouts indicate leverage points: (1) define an MVS floor for “adequate light,” (2) set the postpartum default order-set to be MVS-compliant and require reason-coded step-downs, and (3) implement a minimum indicator set with governance that remains auditable without excessive burden. KPI, key performance indicator; MVS, minimum viable standard.

Together, these mechanisms form a reproducible chain—label vagueness → procurement simplification → menu-cycle narrowing → order-set default → key performance indicators drift—summarized in Figure 1, alongside three leverage points for correction.

## 4 Why low nutrient density is not neutral

In hospital menu design, nutrient density is best conceptualized as “nutrients per calorie”—the amount of protein, essential fats, vitamins, and minerals delivered per unit of energy (12). Here, we use nutrient density to denote composition quality and adequacy to denote total protein–energy delivered; both are required. Operationally, this requires specifying (i) composition targets (e.g., minimum grams of high-quality protein per tray and inclusion of priority micronutrient carriers via foods or fortification) and (ii) adequacy targets (e.g., whether delivered trays plausibly meet protein–energy needs within the inpatient window). This is not a “heavy versus light” argument; it is a composition-and-adequacy argument that can be achieved using culturally acceptable foods.

In postpartum diet research, soups, porridges, and other soft foods are commonly described as components of postpartum eating patterns among Chinese women (8). These items can be clinically appropriate and culturally acceptable, but in hospital settings they may deliver substantial volume without proportional energy or complete protein unless paired with solid, protein-dense foods. The policy concern is displacement: within limited meal opportunities and variable access to snacks, volume-forward trays can crowd out foods that carry most deliverable protein and energy in hospital meals.

Early postpartum recovery, particularly after cesarean birth, is a period in which adequate oral intake supports tissue repair, mobility, and functional reserve. Enhanced Recovery After Surgery-based postoperative care for cesarean delivery supports early oral intake (fluids and food) and timely diet advancement when clinically appropriate, positioning nutrition as part of standardized recovery rather than an optional add-on (5). Surgical nutrition guidance similarly emphasizes avoiding underfeeding and delivering sufficient protein and energy to preserve lean mass and recovery capacity—principles directly relevant to postoperative postpartum patients (13). When trays are persistently low in nutrient density, insufficient intake may plausibly manifest as fatigue or slower functional recovery, even if no single meal appears problematic.

Hydration is essential for maternal well-being, but fluid volume is not a substitute for protein–energy and micronutrient adequacy during lactation and early recovery (14). Guidance for breastfeeding mothers emphasizes overall diet quality and key micronutrients (15). In inpatient contexts, liquids-forward or very low-density menus can therefore appear adequate because fluids are abundant, while still underdelivering protein, energy, and micronutrient carriers. The practical implication is straightforward: soups and porridge can remain components of a “light” pattern, but the default tray requires explicit, deliverable anchors for protein and other nutrient-dense foods.

Low nutrient density is therefore not neutral; it is a modifiable exposure created by defaults in menu design, ordering rules, procurement specifications, and monitoring (4). The next section outlines practical levers and metrics that preserve cultural acceptability while engineering adequate, high-value postpartum trays. Any redesign of postpartum meal defaults must therefore be judged not only by nutrient adequacy on paper, but also by whether mothers accept, consume, and value what is delivered in practice, without avoidable increases in refusal or plate waste.

## 5 Current progress: what is improving and what remains missing

Perioperative nutrition and Enhanced Recovery After Surgery principles are increasingly protocolized, and this logic is now more consistently reflected in cesarean recovery pathways. The 2025 Enhanced Recovery After Surgery society recommendations for postoperative care after cesarean delivery support early oral fluids and feeding, alongside standardized, team-based routines when clinically appropriate (5). The 2025 European Society for Clinical Nutrition and Metabolism surgical nutrition guideline update similarly emphasizes early oral/enteral nutrition when feasible and adequate protein–energy provision to support recovery (13). Collectively, this framing repositions nutrition from an amenity to a deliberately designed clinical component, providing a policy rationale for postpartum wards to specify minimum intake targets while preserving clinically appropriate flexibility (16, 17).

In parallel, hospitals are strengthening the role of dietitians and nutrition leaders in meal design, patient communication, and quality improvement. Implementation-oriented accounts emphasize that durable change is primarily a governance task—aligning clinical intent, kitchen workflows, and accountability—rather than relying on individual effort or “better recipes” alone (18). National standards for healthcare food and drink further demonstrate how organizations can codify expectations for meal quality, access, and monitoring at the system level (9). For postpartum services, the operational implication is to embed dietitian input into menu specifications and escalation rules, rather than treating dietitian involvement as discretionary consultation (18).

Digital ordering and feedback loops represent a second area of momentum because they convert meals into measurable service outputs. Personalization approaches that align menus with patient needs have been associated with reduced food waste and costs during hospitalization, and electronic bedside meal ordering systems have also been associated with higher energy and protein intake, suggesting that operational gains are achievable when tray content better matches patient requirements and preferences (19, 31). When ordering systems routinely capture selections, substitutions, and returns, they can support dashboards for satisfaction, waste, and low-burden intake proxies that enable iterative improvement.

What remains missing is a postpartum-specific minimum standard and quality-governance layer that translates labels such as “light” into nutrient targets, procurement specifications, and ward-level accountability. Where explicit standards exist, they provide a template for defining deliverables and auditing performance rather than relying on informal norms (9). Implementation experience likewise indicates that sustained improvement requires clear ownership, fit-for-purpose metrics, and cross-department coordination between clinical and food-service teams (18). The next section translates this gap into practical, embed-ready solutions—minimum standards, measurable metrics, and accountability mechanisms that can be built into procurement and routine audit cycles.

## 6 System-level responses

### 6.1 Reset the default: “nutritionally adequate light” + minimum viable standards (MVS)

Resetting the default begins by redefining “light” as *nutritionally adequate light*: gentle textures, modest seasoning, and tolerable cooking methods can remain fully compatible with early recovery, but adequacy in energy and protein should not be optional (5, 13). Operationally, postpartum meals should default to an “adequate light” package that meets a defined minimum floor, while more restrictive “step-down” options are treated as explicit exceptions (e.g., intolerance, nausea, clinical restrictions, or informed preference). Defaults matter because they determine what is delivered most frequently (20). Without a defined floor, ambiguity predictably becomes under-delivery (9).

MVS should be defined as the minimum acceptable meal structure that reliably delivers nutrient density without imposing a rigid nutrient prescription, thereby remaining feasible for procurement and ward operations (9). In practice, MVS can be operationalized within menu specifications and ordering rules as a small set of non-negotiables, for example:

- A protein anchor is included in ≥2 eating occasions per day (e.g., egg, tofu/soy, lean meat, dairy).
- A staple base is included at lunch and dinner (e.g., rice, noodles, other staples).
- Vegetables and/or fruit are included daily.
- Soups function as supportive add-ons rather than substitutes for protein- and energy-carrying foods.

This approach standardizes the floor while preserving culturally familiar “light” formats and clinician-directed tailoring when indicated. We operationalize the “nutritionally adequate light” floor as an MVS and summarize its non-negotiables in Table 1 (Panel A). Supplementary Figure S1 visualizes this floor as a four-anchor template and illustrative prototypes for ordering and tray assembly.

**Table 1:** Minimum viable standards (MVS) for a nutritionally adequate postpartum “light diet,” and a minimum auditable indicator set with governance.

Panel A. MVS floor (“adequate light”): non-negotiables			
MVS element	Operational definition (minimum)	Examples (culturally compatible)	Rationale/guardrail
Protein anchor	Protein-carrying item appears in ≥2 eating occasions/day	egg, tofu/soy, lean meat/fish, dairy/fortified soy	Prevents “light” from becoming low-protein by default
Staple base	Staple present at lunch and dinner	rice, noodles, congee + additional staple as needed	Ensures energy-carrying base is not displaced by liquids
Vegetables/fruit	Vegetables and/or fruit daily	cooked greens, soft vegetables, fruit/fruit portions	Maintains micronutrient/fiber carriers within “light” framing
Soup as add-on	Soups are supportive add-ons, not substitutes for protein/energy carriers	soup alongside protein + staple	Prevents “soup-only” trays from meeting the label

Panel B. Minimum indicator set + governance (auditable, not burdensome)			
Indicator	Definition (what is counted)	Low-burden data source	How it is used (governance action)
Default integrity	An MVS-compliant postpartum set exists and is the pre-selected option	ordering interface/order set configuration	Quarterly (or when menus change) verify default remains MVS-compliant; document changes
MVS compliance rate	Share of delivered trays that meet the MVS checklist	simple tray-level checklist (4 items from Panel A)	Review monthly/bi-monthly; if drift occurs, adjust menu cycle or assembly rules
Exception rate + reason	Rate of step-downs to more restricted diets, with a reason field captured	order modification log + reason codes	Exceptions become reviewable “safety” data; identify preventable step-downs
Intake proxy	Low-burden consumption/plate-waste proxy (e.g., mostly/partly eaten)	routine tray return note/quick tick box	Signals acceptability/feasibility; triggers targeted menu tweaks
Patient experience	Brief measure of perceived adequacy (hunger/recovery) + clarity of options	2–3 item short tool where feasible	Identifies “adequate on paper but not in experience”; informs counseling scripts
Governance (owner group)	Named interdisciplinary owners + review cadence + audit trail	standing huddle/QI log	Keeps accountability explicit; prevents drift back to ambiguous “light” labels

“Panel A defines the floor for ‘adequate light’ (addressing label vagueness), while Panel B makes the floor auditable and prevents KPI drift by tracking defaults, compliance, exceptions, intake, and experience.” Conceptual framework proposed by the authors.

MVS, minimum viable standard(s); KPI, key performance indicator.

### 6.2 Minimum indicator set + governance (keep it auditable, not burdensome)

An MVS policy is actionable only if paired with a small, routine indicator set that makes “adequate light” visible to clinical leaders and food services (9). To keep this Viewpoint operational rather than encyclopedic, we propose a minimum set that can be captured through routine delivery and ordering processes (not bespoke audits) (20, 21).

1. Default integrity: an MVS-compliant postpartum set exists and is the pre-selected option (9).
2. MVS compliance rate: proportion of delivered trays meeting MVS (via a simple tray-level checklist). Supplementary Table S1 provides the tray-level MVS checklist and data dictionary used to define MVS compliance (including allowed substitutions and missingness rules).
3. Exception rate and reasons: downgrades to more restricted diets are recorded with a reason field (turning “safety” into reviewable data). To standardize exception documentation and enable auditable review, Supplementary Table S2 specifies a minimal set of reason codes for step-down orders.
4. Intake proxy: a low-burden consumption/plate-waste proxy (e.g., mostly/partly eaten) (19).
5. Patient experience: perceived adequacy for hunger/recovery and clarity of options, using a brief tool where feasible (22). Supplementary Table S3 proposes a minimal 3–5 item patient-experience set and a simple ‘mostly/partly eaten’ intake proxy to support routine monitoring with minimal burden.

Table 1 (Panel B) translates this approach into a minimum auditable indicator set—capturable through routine ordering and tray-delivery processes—paired with a light governance loop to prevent drift.

Supplementary Figure S2 maps where these indicators can be captured within routine workflows and how the owner-group review closes the loop.

Accountability should sit with a small interdisciplinary owner group, with an obstetric service lead and a dietitian lead serving as formal co-owners of postpartum menu specifications, exception governance, and routine dashboard review. This group should approve menu changes, oversee exception pathways, and maintain a brief decision log to prevent drift from “light” to nutritionally underpowered defaults (9, 18). Improvement can follow short learning cycles and scale once stable (23). Where catering is outsourced, the MVS floor and reporting cadence should be embedded as contract deliverables to prevent reversion to ambiguous “light” labels (24).

## 7 Discussion

### 7.1 Synthesis

The central claim of this Viewpoint is that postpartum hospital meals should be engineered to deliver adequate nutrient density through well-governed defaults, rather than framed as a binary choice between “light” and “non-light.” Gentle textures and modest seasoning can be fully compatible with early recovery when paired with sufficient energy and protein, and when exceptions are explicitly indicated rather than implicitly assumed. This framing does not reduce postpartum care to food alone; rather, it recognizes that meal defaults operate within a brief, high-demand period in which mothers are simultaneously recovering, bonding, and establishing feeding routines. Enhanced recovery guidance for cesarean delivery supports early oral intake and standardized recovery pathways when clinically appropriate, reinforcing that nutrition belongs within recovery design rather than outside it (5). The practical implication is that postpartum wards can preserve culturally familiar “light” formats while tightening the minimum adequacy floor.

A system framing is essential because the “light-diet default” is produced by interacting incentives—risk minimization, operational simplicity, and standardization—so correction requires redesigning the package rather than persuading individuals. Treating meals as care infrastructure aligns with Food is Medicine arguments that call for integrating nutrition into healthcare delivery rather than relegating it to hospitality (4). This approach also positions nutrition security and access to appropriate food as health-system responsibilities, legitimizing the use of standards, performance measurement, and iterative service improvement (25–27). Under this lens, acceptability and safety are not competing aims; they can be co-designed with adequacy when menu specifications and ordering logic are explicit. A nutritionally stronger default that mothers do not want to eat would be self-defeating if it increases refusal or waste and reduces actual intake.

### 7.2 Discussion—future directions

Research should begin with baseline mapping that makes postpartum defaults visible, using mixed-methods approaches that are low-burden and operationally interpretable. A pragmatic starting design is a menu audit linked to tray-level proxies (e.g., presence of protein-bearing items and staples), paired with plate-waste or consumption proxies and brief patient interviews to explain acceptability and constraint pathways (9). Because defaults act through ordering environments, evaluations should explicitly document what is pre-selected, what is optional, and what triggers exceptions (28). Where feasible, hospitals can embed these measures within routine quality improvement evaluation, using stepped implementation across wards or interrupted time series analyses to assess whether default changes improve MVS compliance and perceived adequacy without unintended increases in intolerance-related exceptions (28–30).

Practice improvement should prioritize integration rather than add-on counseling. Dietitians can be embedded into postpartum order sets, exception pathways, and periodic ward rounds to align clinical intent with what is delivered at the tray level (9). Existing recovery pathways legitimize early intake and structured postoperative routines and can be adapted to postpartum contexts without assuming identical physiology or risk profiles (5). Implementation should also address family-facing scripts by reframing “light” as “light but adequate,” using shared decision-making language that respects preferences while offering a nutritionally complete default. Digital ordering prompts can surface protein options, clarify portion expectations, and require a reason field when stepping down to more restricted diets, turning variability into reviewable data rather than silent drift (28).

Policy directions should focus on governance levers that are compatible with hospital operations. Postpartum meal quality can be incorporated into maternal-care quality governance as a small set of routine indicators with periodic reporting and review, rather than one-off campaigns (9). Procurement specifications and catering requirements can then be aligned to those standards, including menu deliverables, audit rights, reporting cadence, and corrective-action timelines that keep defaults stable over time (9). A Food is Medicine framing provides a policy rationale by treating food delivery as a health intervention with accountable infrastructure rather than discretionary comfort provision (25).

### 7.3 Author viewpoint

Postpartum patients have limited bargaining power within hospital food systems; adequacy must therefore be designed into the default rather than negotiated at the bedside. Success is measurable: routine reporting of MVS compliance, a brief patient-reported measure of adequacy and option clarity, and auditable exceptions with documented reasons and review.

## Data Availability

The original contributions presented in the study are included in the article/Supplementary material, further inquiries can be directed to the corresponding author.

## Supplementary material

SUPPLEMENTARY FIGURE S1 “Nutritionally adequate light” prototypes (MVS floor operationalization). Panel **(A)** presents a four-anchor template for an “adequate light” postpartum tray—staple base, protein anchor, vegetables/fruit carrier, and soup as an add-on rather than a substitute—and highlights common failure modes when “light” is treated as a permissive label. Panels **(B–D)** provide illustrative prototype tray structures (e.g., congee-based, rice + soup menu-set, and noodle-based variants) that preserve culturally familiar “light” formats while ensuring the anchors remain present. These prototypes are intended as implementation aids for menu design and ordering defaults, not as patient-specific prescriptions. MVS, minimum viable standard.

SUPPLEMENTARY FIGURE S2 Low-burden workflow to capture the minimum indicator set and close the governance loop (MVS policy). **(A)** Maps where each indicator can be captured within routine ordering, tray assembly/delivery, and brief bedside feedback processes, avoiding bespoke audits. **(B)** Provides an illustrative reason-code structure for step-down orders, converting “safety exceptions” into reviewable data. S2C depicts a light governance loop in which an interdisciplinary owner group reviews a small dashboard, approves menu/default changes, and maintains an auditable decision log to prevent drift back to ambiguous “light” labels. MVS, minimum viable standard; KPI, key performance indicator.
