# Supplementary figures and images for: Correcting the “light-diet default”: nutrient density gaps in hospital-based postpartum nutrition services in China and system-level responses
Source: Front Public Health. 2026 Apr 10;14:1769297. doi: 10.3389/fpubh.2026.1769297 (PMC13105905; doi:10.3389/fpubh.2026.1769297)

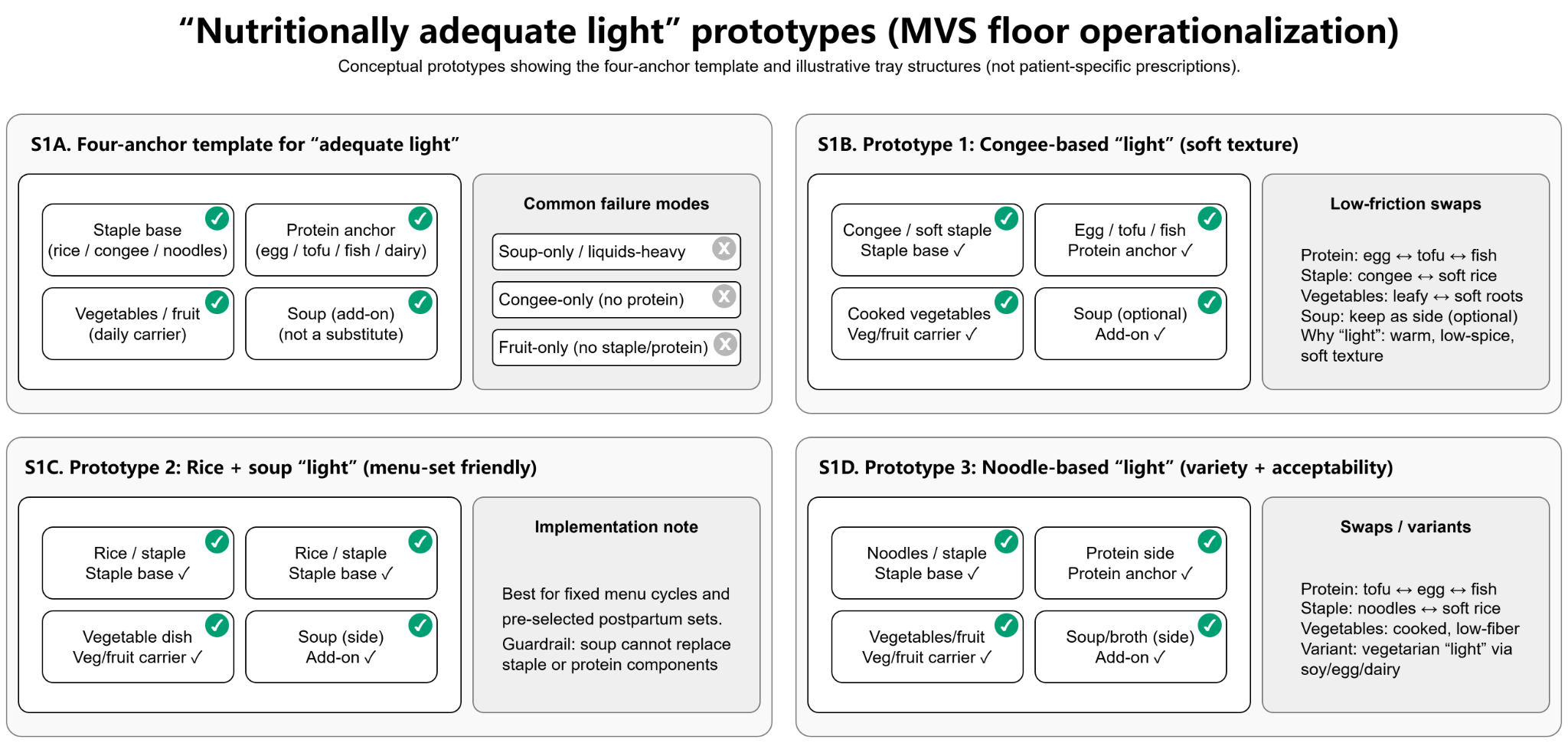

Supplement: Supplementary file 1 [file Image_1.TIFF]

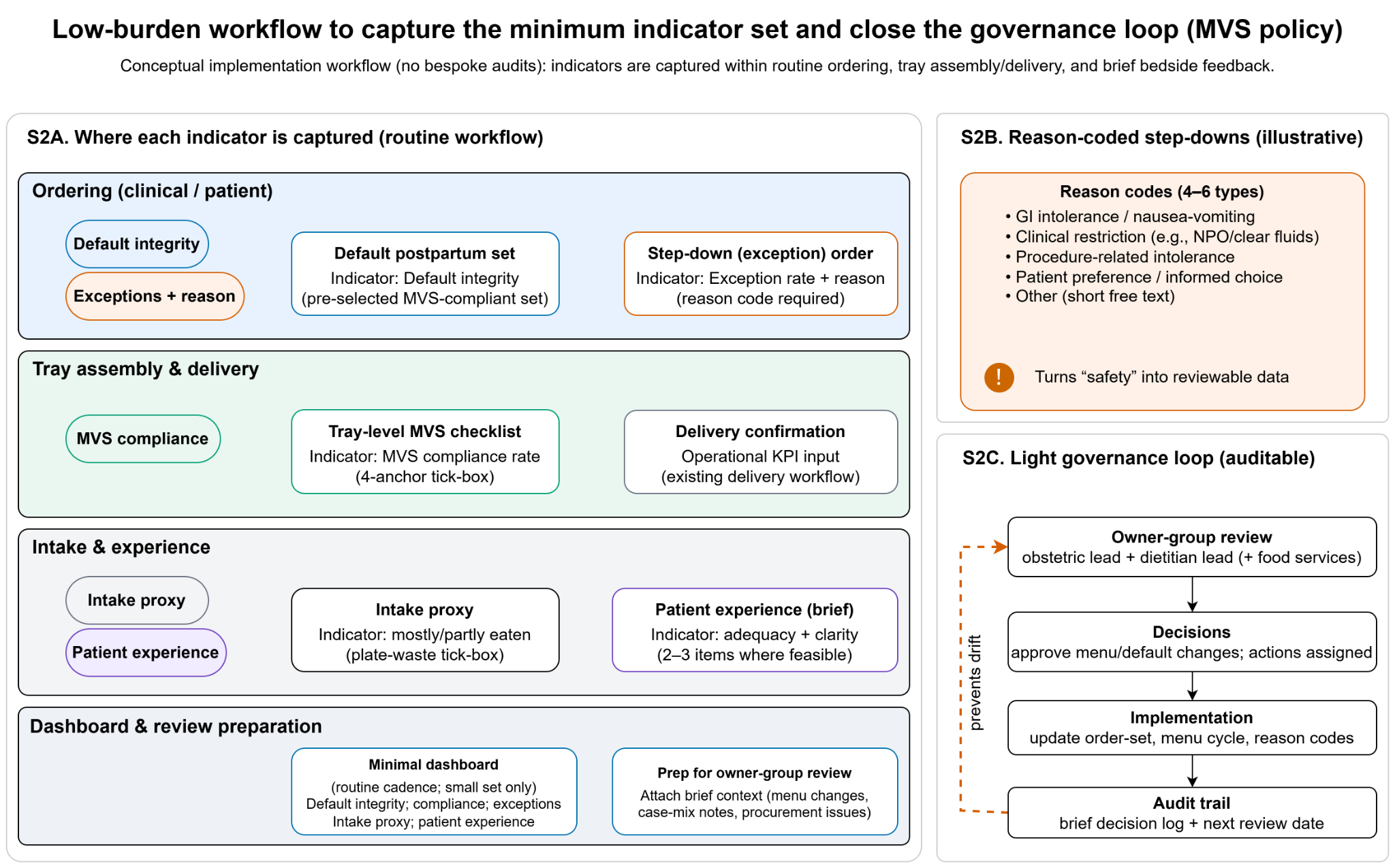

Supplement: Supplementary file 2 [file Image_2.TIFF]
