# Supplementary material for: Correcting the “light-diet default”: nutrient density gaps in hospital-based postpartum nutrition services in China and system-level responses
Source: Front Public Health. 2026 Apr 10;14:1769297. doi: 10.3389/fpubh.2026.1769297 (PMC13105905; doi:10.3389/fpubh.2026.1769297)
Supplement: Supplementary file 3 [file Table_1.DOCX]

**Supplementary Table S1. Tray-level MVS compliance checklist and data dictionary (for calculating “MVS compliance rate”)**

**Supplementary Table S1A. Tray-level MVS compliance checklist (4 items from Table 1, Panel A)**

| **Section** | **Field** | **Response options** | **Operational definition (minimum)** | **Examples (aligned with Table 1 Panel A)** | **Notes / guardrails** |
| --- | --- | --- | --- | --- | --- |
| Identification | date | YYYY-MM-DD | Date of tray delivery | — | — |
| Identification | meal_type | Breakfast / Lunch / Dinner / Snack | Meal occasion for this tray | — | Staple requirement applies to **Lunch/Dinner** only |
| Exceptions | step_down_flag | 0/1 | Whether tray reflects a step-down (exception) from the default MVS-compliant set | — | If 1, reason_code is required |
| Exceptions | reason_code | GI intolerance/N&V; Clinical restriction (e.g., NPO/clear fluids); Procedure-related intolerance; Patient preference/informed choice; Other | Reason-coded step-down (turns “safety” into reviewable data) | — | Required if step_down_flag=1 |
| Anchor 1 | protein_anchor_present | Y / N / UNK | A clearly identifiable protein-carrying item is present as a distinct component (not garnish) | egg; tofu/soy; lean meat/fish; dairy/fortified soy | UNK if tray contents not observable/recorded |
| Anchor 1 (optional detail) | protein_anchor_type | Egg; Tofu/soy; Fish; Lean meat/poultry; Dairy/fortified soy; Mixed dish; Other; UNK | Main protein carrier type | as at left | “Mixed dish” allowed if protein component is clear |
| Anchor 2 | staple_base_present | Y / N / NA / UNK | Staple base present at lunch and dinner | rice; noodles; congee (+ additional staple as needed) | Set NA for Breakfast/Snack unless your local rule requires |
| Anchor 2 (optional detail) | staple_type | Rice; Noodles; Congee; Bread/steamed bun; Other; UNK | Staple category | as at left | Optional; useful for procurement/menu-cycle review |
| Anchor 3 | vegfruit_present | Y / N / UNK | Vegetables and/or fruit present as a recognizable component | cooked greens; soft vegetables; fruit/fruit portions | Garnish-only does not count |
| Anchor 3 (optional detail) | vegfruit_type | Cooked greens; Soft vegetables; Fruit; Mixed; Other; UNK | Vegetable/fruit category | as at left | Optional |
| Anchor 4 | soup_present | Y / N / UNK | Any soup/broth provided | soup alongside protein + staple | If N → soup_role=NA |
| Anchor 4 | soup_role | Add-on / Substitute / NA / UNK | Add-on = supportive side; Substitute = displaces protein/energy carriers | “Add-on” when protein + staple still present | Substitute flags “soup-only / liquids-heavy” trays |
| Derived (auto) | mvs_tray_pass | Y / N / UNK | Derived per Supplementary Table S1B scoring rules | — | Do not hand-score; derive automatically |

**Supplementary Table S1B. Data dictionary and scoring rules for “MVS compliance rate”**

| **Variable** | **Definition (what is counted)** | **Allowed values / coding** | **Decision rule** | **Missingness rule** |
| --- | --- | --- | --- | --- |
| meal_type | Meal occasion for the delivered tray | Breakfast/Lunch/Dinner/Snack | Staple base required only for Lunch/Dinner | If missing → UNK; exclude from tray-level scoring |
| step_down_flag | Step-down (exception) from the default MVS-compliant postpartum set | 0=No; 1=Yes | If 1, reason_code required | If 1 and reason_code missing → data quality flag |
| reason_code | Reason-coded step-down category | GI intolerance/N&V; Clinical restriction (e.g., NPO/clear fluids); Procedure-related intolerance; Patient preference/informed choice; Other | Used to compute “Exception rate + reason” and audit preventable step-downs | NA if step_down_flag=0; otherwise, must not be UNK |
| protein_anchor_present | Presence of a protein-carrying item on the tray | Y/N/UNK | Counts only when a distinct protein-carrying item is present | If UNK → mvs_tray_pass=UNK |
| protein_anchor_type (optional) | Type of protein carrier | Egg; Tofu/soy; Fish; Lean meat/poultry; Dairy/fortified soy; Mixed; Other; UNK | Not used in pass/fail; supports menu-cycle review | Optional; UNK allowed |
| staple_base_present | Presence of a staple base on the tray | Y/N/NA/UNK | Required if meal_type is Lunch/Dinner; NA otherwise | If UNK when required → mvs_tray_pass=UNK |
| vegfruit_present | Vegetables and/or fruit present on the tray | Y/N/UNK | Recognizable component; garnish-only does not count | If UNK → mvs_tray_pass=UNK |
| soup_present | Soup/broth provided | Y/N/UNK | If N → soup_role=NA | If UNK → mvs_tray_pass=UNK only if soup_role needed |
| soup_role | Whether soup functions as add-on vs substitute | Add-on/Substitute/NA/UNK | Substitute if tray is “soup-only/liquids-heavy” and protein and/or staple base is missing | If UNK and soup_present=Y → mvs_tray_pass=UNK |
| mvs_tray_pass (derived) | Tray-level MVS checklist pass/fail | Y/N/UNK | Lunch/Dinner pass = protein_anchor_present=Y AND staple_base_present=Y AND vegfruit_present=Y AND soup_role≠Substitute. Breakfast/Snack (optional) pass = protein_anchor_present=Y AND soup_role≠Substitute (staple=NA). | If any required component is UNK → mvs_tray_pass=UNK |
| MVS compliance rate (indicator) | Share of delivered trays meeting the MVS checklist (Table 1 Panel B) | % | Numerator: count(mvs_tray_pass=Y). Denominator (recommended): eligible trays with complete data (exclude UNK) and report completeness separately. | Also report: data completeness rate; UNK rate by anchor |

**Table note.** The tray-level MVS checklist operationalizes the four non-negotiables in Table 1 (Panel A)—protein anchor, staple base (lunch/dinner), vegetables/fruit, and soups as add-ons (not substitutes)—to provide a reusable definition for calculating the “MVS compliance rate” (Table 1, Panel B). Items are recorded as tick-box fields at the point of tray assembly/delivery; when any required component is not observable, it is coded as UNK and excluded from pass/fail scoring, with completeness reported separately.

**Abbreviations:** MVS, minimum viable standard(s); KPI, key performance indicator; NPO, nil per os; UNK, unknown/not observable.
