# Supplementary material for: Correcting the “light-diet default”: nutrient density gaps in hospital-based postpartum nutrition services in China and system-level responses
Source: Front Public Health. 2026 Apr 10;14:1769297. doi: 10.3389/fpubh.2026.1769297 (PMC13105905; doi:10.3389/fpubh.2026.1769297)
Supplement: Supplementary file 4 [file Table_2.DOCX]

**Supplementary Table S2. Standardized reason codes for step-down (exception) orders**

| **Reason code (dropdown label)** | **Code** | **When to use (operational trigger)** | **Examples (typical scenarios)** | **Exclusion / guardrail** | **Optional fields** |
| --- | --- | --- | --- | --- | --- |
| Nausea/vomiting | 01 | Patient has active nausea/vomiting or strong emesis risk at the time of ordering | postoperative nausea; vomiting after oral intake; antiemetic being titrated | Do not use for “general caution” without symptoms | symptom_severity (mild/moderate/severe); onset_time (optional) |
| GI intolerance (bloating/diarrhea/abdominal pain) | 02 | Documented GI intolerance that limits intake (non-vomiting) | abdominal distension; diarrhea; cramping; reflux/heartburn affecting intake | Must reflect a current intolerance, not historical preference | main_symptom (dropdown); duration (optional) |
| Clinical restriction: NPO/clear fluids for procedure/exam | 03 | Formal restriction due to procedure/exam/clinical order requiring NPO or clear fluids | imaging/OR/endo; anesthesia-related NPO; fasting for labs | Should match an order or documented restriction window | restriction_type (NPO/clear fluids); expected_end_time |
| Postoperative or condition-specific diet restriction (non-NPO) | 04 | Clinically indicated diet modification beyond “light,” but not full NPO | e.g., strict low-residue per clinician; fluid restriction; renal restriction | Do not use if reason is purely operational (supply) | restriction_spec (short text) |
| Pain/fatigue limiting intake (temporary) | 05 | Intake is limited primarily by pain, fatigue, sedation | severe incision pain; excessive drowsiness | Should be temporary and reviewable | analgesia_adjusted (Y/N) |
| Patient preference / informed choice | 06 | Patient explicitly prefers a more restrictive option after being informed of default | requests soup-only; declines protein item; cultural/personal preference | Not for “assumed preference”; must be expressed | preference_type (dropdown); education_given (Y/N) |
| Allergy/intolerance to a component requiring substitution | 07 | Step-down occurs because safe substitution not available at time of ordering | allergy to egg/dairy/soy; intolerance to specific item | Prefer swap within MVS rather than step-down when feasible | allergen (dropdown); swap_attempted (Y/N) |
| Operational constraint / supply issue | 08 | Default MVS tray cannot be delivered due to supply/logistics | protein item out of stock; delivery delay causing omission | Critical guardrail: this is preventable drift; triggers procurement action | affected_item (dropdown); incident_flag (Y/N) |
| Other (must specify) | 99 | None of the above fits; requires explanation | unusual clinical context | Mandatory reason_text; reviewed in governance meeting | reason_text (required); reviewer_flag |

**Table note**

- Reason codes are required whenever a “step-down” order is placed (step_down_flag=1).
- Codes 08 (Operational constraint) and 99 (Other) should be flagged for review to prevent drift back to ambiguous “light” defaults and to identify avoidable system failures.

**Abbreviations:** MVS, minimum viable standard(s); NPO, nil per os.
