# Supplementary material for: Correcting the “light-diet default”: nutrient density gaps in hospital-based postpartum nutrition services in China and system-level responses
Source: Front Public Health. 2026 Apr 10;14:1769297. doi: 10.3389/fpubh.2026.1769297 (PMC13105905; doi:10.3389/fpubh.2026.1769297)
Supplement: Supplementary file 5 [file Table_3.DOCX]

**Supplementary Table S3. Minimal patient experience items and intake proxy rules (low-burden tool)**

**S3A. Patient experience mini-scale (3–5 items; 5-point Likert)**

**Stem:** “Thinking about today’s postpartum meals…”

| **Item ID** | **Item (English, copy-ready)** | **Construct** | **Response options (0–4)** | **Rationale (why it matters for governance)** |
| --- | --- | --- | --- | --- |
| PX1 | “The meals felt adequate for my hunger and recovery needs.” | Perceived adequacy | 0=Strongly disagree … 4=Strongly agree | Captures “adequate on paper but not in experience.” |
| PX2 | “The meal options and labels (e.g., ‘light’, ‘step-down’) were clear to me.” | Clarity of options | 0–4 | Detects confusion-driven under-selection or unnecessary restriction. |
| PX3 | “It was easy to obtain protein-containing items (e.g., egg/tofu/fish/dairy) when I wanted them.” | Access to protein | 0–4 | Directly maps to the MVS “protein anchor” logic and operational access barriers. |
| PX4 (optional) | “The meals were acceptable to eat (taste/texture/temperature).” | Acceptability | 0–4 | Helps distinguish adequacy vs palatability constraints driving waste. |
| PX5 (optional) | “I could request changes (swap items) without difficulty when needed.” | Flexibility / responsiveness | 0–4 | Identifies whether “low-dispute defaults” are suppressing patient-tailored swaps. |

Recommended minimum set: PX1–PX3 (3 items).

If you can afford 4–5 items: add PX4 and/or PX5.

Scoring (recommended for low-burden reporting):

PX_total_mean = mean of answered items (range 0–4).

PX_flag_low = 1 if PX1 ≤2 (neutral or below) OR PX_total_mean <2.5 (site chooses threshold).

Report monthly: mean score + % flagged low.

S3B. Intake proxy (plate-waste tick-box) — recording rule

| **Category** | **Code** | **Definition (operational)** | **Recording rule (low-burden)** |
| --- | --- | --- | --- |
| Mostly eaten | 2 | ~≥75% of tray contents consumed | Mark at tray return, or patient reports “ate most.” |
| Partly eaten | 1 | ~25–74% consumed | Use when intake clearly partial. |
| Hardly/none | 0 | <25% consumed or not eaten | Use when tray largely untouched. |
| Not observed / not applicable | 9 | Tray not returned/unknown; patient away for exam | Avoid guessing; code 9 and track completeness. |

**Guardrails**

- Record **overall tray intake**, not each component (keeps burden low).
- If a patient ate only soup/fruit and left protein/staple untouched, still code based on overall proportion; if you want more sensitivity, add the optional item below.

**Optional add-on (if feasible, 1 extra tick-box):**
(Y/N/UNK) — “Was the main protein item left uneaten?”
Purpose: identifies “protein access/acceptability” issues even when overall intake seems moderate.

**S3C. Data dictionary**

| **Variable** | **Definition** | **Values** |
| --- | --- | --- |
| respondent_type | Who answered | patient / proxy / nurse observation |
| PX1–PX3 (±PX4–PX5) | Experience items | 0–4 (Strongly disagree → Strongly agree) |
| PX_total_mean | Mean of answered items | 0–4 |
| intake_proxy | Plate-waste proxy | 0/1/2/9 |
| completeness_flag | Data completeness for dashboard | complete / incomplete |

**Table note (for Supplementary Table S3)**

This minimal tool provides a low-burden complement to the tray-level MVS checklist by capturing (i) a brief patient-reported experience signal (adequacy, clarity, and access to protein) and (ii) a simple intake proxy (“mostly/partly eaten”). Together, these measures support the “Patient experience” and “Intake proxy” indicators in Table 1 (Panel B) and help distinguish nutrient-design issues from acceptability and implementation barriers.

**Abbreviations**

MVS, minimum viable standard(s); KPI, key performance indicator; PX, patient experience; UNK, unknown/not observed; NPO, nil per os.
